# Alogliptin Mitigates Methotrexate-Induced Nephrotoxicity in a Rat Model: Antagonizing Oxidative Stress, Inflammation and Apoptosis

**DOI:** 10.3390/ijms26199608

**Published:** 2025-10-01

**Authors:** Marwa M. Fahmy, Heba A. Habib, Esraa M. Zeidan, Yousef A. Bin Jardan, Gehan H. Heeba

**Affiliations:** 1 Minia University Hospital, Minia University, Minia 61519, Egypt; marwa.mohsen@pharm.s-mu.edu.eg; 2 Department of Pharmacology and Toxicology, Faculty of Pharmacy, Minia University, Minia 61519, Egypt; ph_esraamohammed@mu.edu.eg; 3 Department of Pharmaceutics, College of Pharmacy, King Saud University, Riyadh 11451, Saudi Arabia; ybinjardan@ksu.edu.sa

**Keywords:** methotrexate, nephrotoxicity, alogliptin, Nrf2, TNF-α, c-caspase-3

## Abstract

Although methotrexate (MTX) is a magnificent cure for cancerous neoplasms and inflammatory disorders, its usage is bound due to associated hazards, especially nephrotoxicity. The present study investigated the possible therapeutic impact of alogliptin (ALO), prescribed for managing type 2 diabetes, on renal injury caused by MTX and explored the mechanisms that could illustrate this suggested protective effect. Four rat groups were involved: control, ALO (20 mg/kg/d, intragastrically (I.G.)) for ten days, MTX, and MTX + ALO groups. The latter two groups were given MTX (20 mg/kg, I.P.) on the 7th day, while the MTX + ALO group was administered ten days of 20 mg/kg of ALO. A significant impairment in renal function, catalase activity, reduced glutathione content, nuclear factor erythroid 2-related factor 2 (Nrf2), and heme oxygenase-1 (HO-1) expressions, coupled with an increase in kidney injury molecule-1 (KIM-1), malondialdehyde, tumor necrosis factor-alpha (TNF-α), and cleaved caspase-3 (c-caspase-3) expressions, was observed in MTX-intoxicated rats, evidenced by remarkable deterioration in renal construction. Conversely, ALO improved renal function and architecture. Moreover, ALO retrieved the oxidative balance, the attenuated Nrf2/HO-1 expression, and the elevated KIM-1, TNF-α, and c-caspase-3 expression. In conclusion, ALO might abrogate MTX-elicited kidney damage by rectifying the deviation in oxidative status, apoptotic and inflammatory pathways, paving the way for managing MTX-induced nephrotoxicity.

## 1. Introduction

Methotrexate (MTX) is an effective anti-folate medication used at high doses for managing various malignancies [1] and at low doses for treating non-malignant autoimmune and inflammatory-related disorders [2]. Nevertheless, the threat of nephrotoxicity associated with its administration is a fundamental dilemma that puts boundaries on its clinical utility [3,4]. MTX-induced renal injury can range from mild subclinical tubulopathy to acute kidney injury (AKI) [5]. Unfortunately, previous studies have reported the incidence of AKI in about 2–12% of patients following MTX treatment. These studies also spotlighted the interplay between MTX renal hazards and risk of mortality [6,7].

Although there is no precise mechanism mediating the pathogenesis of MTX nephrotoxicity, accumulation of both the parent drug and its metabolite, 7-hydroxy MTX, in renal tubules of damaged kidneys induced by MTX is involved in MTX-provoked renal damage. This accumulation, in a vicious cycle, worsens MTX’s negative impacts [8]. Oxidative injury, infiltration of immune cells, inflammation, and apoptosis are contributing to MTX renal toxicity [9,10]. NADPH oxidase activation and massive reactive oxygen species (ROS) generation [9,11], alongside disruption of the protective pathway mediated by nuclear factor erythroid 2-related factor 2 (Nrf2) [12], have been revealed in earlier studies. These alterations lead to oxidative imbalance, which eventually provokes inflammatory pathways generating tumor necrosis factor-alpha (TNF-α) [13].

Alogliptin (ALO), a dipeptidyl peptidase 4 (DPP-4) inhibitor, is a crucial medication for type-2 diabetic patients. The enzyme DPP-4, a serine peptidase enzyme, inactivates glucagon-like peptide 1 (GLP-1). Hindrance of this enzyme results in a raised GLP-1 level and elongates its effect, which subsequently triggers insulin secretion based on glycemic level without the incidence of hypoglycemic risk [14,15].

Although DPP-4 has been indicated throughout many organs [16], the renal DPP-4 activity per tissue gram is the highest [17]. Changes in DPP-4 levels were observed in pathological kidney settings [17]. Further, DPP-4 was expressed in the human glomerulus in pathological conditions of the kidneys [18]. Exposure of human glomerular epithelial [19] and endothelial [20] cells to interferon-γ and high glucose concentrations, respectively, increased expression of DPP-4. Moreover, a remarkably higher activity of DPP-4 in urine was observed in T2DM individuals with albuminuria than those without albuminuria [21,22]. Wolke, Teumer [23] has reported the inverse interplay between DPP-4 and eGFR. Earlier studies have also pointed to the crosstalk between the development of kidney disease, either diabetic or non-diabetic, and elevated DPP-4 activity [24,25]. These findings support the link between the pathological role of DPP-4 and impaired renal function.

Interestingly, the ability of DPP-4 inhibitors to mitigate apoptosis, inflammation, and oxidative stress has been reported in many studies [26,27]. These outcomes opened the gates for DPP-4 inhibitors to prove their efficacy in protection against kidney injury in diabetic clinical [28,29] and experimental studies [30]. Moreover, DPP-4 deficient rats were protected against renal injury caused by ischemia–reperfusion injury [31]. Nephroprotective influence of DPP-4 inhibitors, independent of controlling blood glucose level, has been witnessed in different animal models of nephrotoxicity induced by cisplatin [32,33], cyclophosphamide [34] and doxorubicin [35,36,37]. Recently, Afkhami Fard, Malekinejad [38] highlighted the nephroprotective impact of sitaglipitin against MTX-produced renal damage. Consequently, this attracts our interest to examine the protective role of ALO in MTX-induced nephrotoxicity.

Despite these findings, nothing is known, until now, regarding the role of ALO in MTX-induced kidney damage. Therefore, this work was performed to assess ALO’s therapeutic potential in reducing renal toxicity caused by MTX and understand the contributed mechanisms.

## 2. Results

### 2.1. Effect of ALO on Glycemic Level, Renal Index, Serum Creatinine and Urea Concentration Besides mRNA Expression of KIM-1 in MTX-Induced Renal Damage

As listed in Table 1, there was no significant difference in random blood glucose levels. However, rats exposed to MTX had notably higher levels of kidney index, serum creatinine, and urea than those of the control group. Conversely, rats pretreated with ALO experienced significant reductions in these three parameters relative to the values obtained from rats receiving MTX alone.

**Figure 1:**
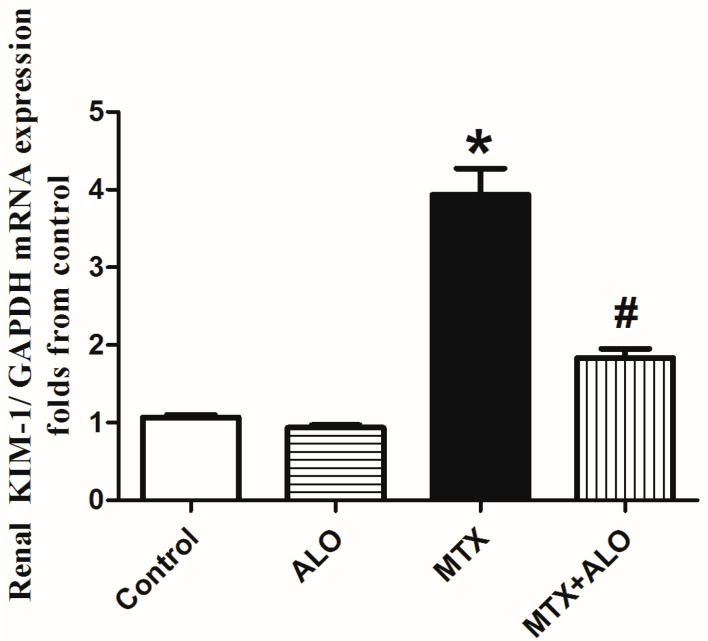
ALO (20 mg/kg) effect on KIM-1 mRNA expression in MTX-induced renal injury rat model. * and # mean a significant (*p* ≤ 0.05) difference from the control group and MTX group, respectively, at *p* ≤ 0.05. ALO: Alogliptin, MTX: Methotrexate, KIM-1: Kidney injury molecule-1.

As presented in Figure 1, the MTX-intoxicated group exhibited significant elevations in mRNA expression of kidney injury molecule-1 (KIM-1) relative to the control rats, pointing to MTX harmful effects on the proximal tubules. The pretreatment with ALO (20mg/kg) significantly ameliorated this increase.

### 2.2. Histopathological Findings of Renal Tissues

**Figure 2:**
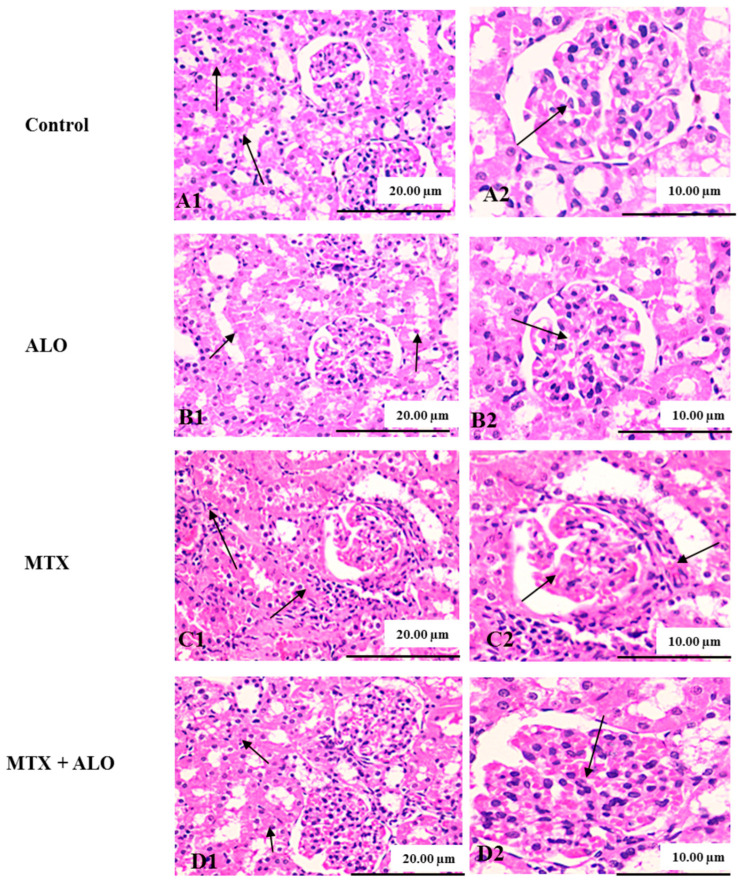
ALO (20 mg/kg) effect on renal tissues stained with H&E of MTX-treated rats. Photomicrograph of control and ALO groups’ renal sections exhibiting intact histological structure of renal tubular epithelial lining (arrow, (**A1**,**B1**), ×200) and intact circumscribed glomerulus with normal Bowman’s capsule (arrow, (**A2**,**B2**), H&E, ×400). Renal tissues of MTX-treated rats presented necrotic epithelial lining of tubules alongside infiltrating mononuclear cells (arrow, (**C1**), ×200) and retracted capillary tufts with thickening and proliferation of Bowman’s capsule forming a crescent-like shape (arrow, (**C2**), H&E, ×400). Photomicrograph of kidney obtained from the MTX + ALO group showing mild tubular epithelial swelling with loss of brush border (arrow, (**D1**), ×200) and mild thickening of Bowman’s capsule with congested hypercellular glomerular tufts (arrow, (**D2**), H&E, ×400). ALO: Alogliptin, MTX: Methotrexate.

Kidney tissue sections acquired from the control and ALO groups revealed normal morphological structure: normal glomerulus with a thin-walled Bowman’s capsule and intact renal tubular epithelial lining. Neither glomerular tuft retraction nor interstitial damage within the interstitial compartment could be observed in these sections (Figure 2A1,A2,B1,B2).

Meanwhile, renal sections obtained from the MTX-treated group exhibited progressive histological aberrations. Capillary tuft retraction and proliferation of the Bowman capsule led to the appearance of the glomeruli with crescent-like features. Moreover, intratubular albuminous casts and necrosis of the epithelial lining are observed. Furthermore, the renal cortex of the MTX group showed blood capillary congestion and mononuclear cell infiltration, particularly lymphocytes and macrophages, in the interstitial compartment (Figure 2C1,C2).

Interestingly, histological abnormalities in endothelial, glomerular, and tubular components were markedly corrected by oral administration of ALO for ten days (Figure 2D1,D2). Renal tissue alterations in the four studied groups had scores that were summarized in Table 2.

### 2.3. Effect of ALO on Renal MDA, GSH, and Catalase in MTX-Induced Renal Damage

**Figure 3:**
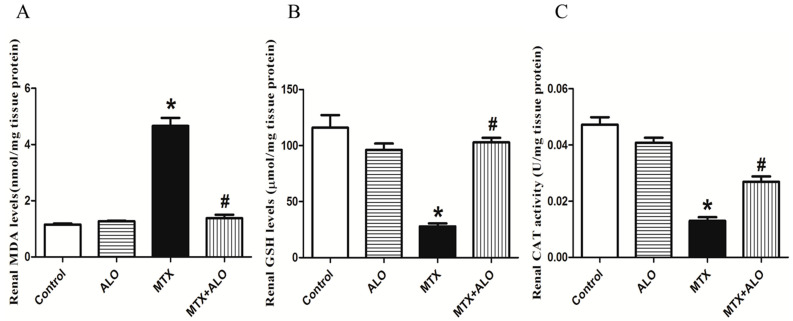
ALO (20 mg/kg) influence on oxidative parameters: (**A**) MDA, (**B**) GSH, and (**C**) Catalase in renal tissues in the MTX-induced renal injury rat model. Findings are displayed as means ± SEM (*n* = 6). * and # mean a significant (*p* ≤ 0.05) difference from the control group and MTX group, respectively, at *p* ≤ 0.05. ALO: Alogliptin, MTX: Methotrexate, MDA: Malondialdehyde, GSH: Reduced glutathione.

It was found that MTX-intoxicated rats exhibited a robust elevation in renal malondialdehyde (MDA) content compared to the control group. However, the MTX + ALO group experienced significant attenuation in MDA levels (Figure 3A).

Consistent with MDA results, the levels of reduced glutathione (GSH) and catalase activities were significantly lower in the MTX group. Conversely, pretreatment with ALO led to a significant elevation in these antioxidant markers (Figure 3B,C).

### 2.4. Effect of ALO on the Renal Nrf2/HO-1 Protein Expression

**Figure 4:**
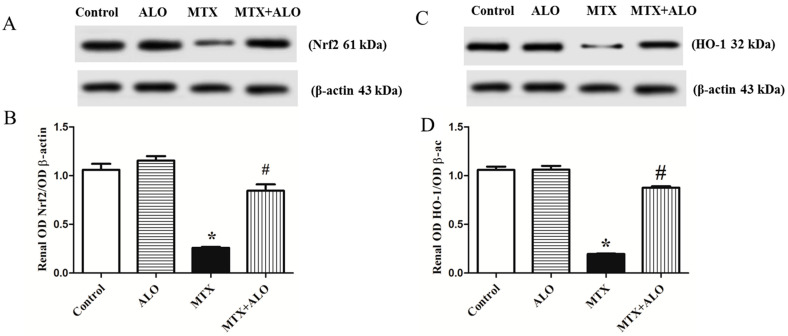
Representative Western blot illustrating the influence of ALO (20 mg/kg) on renal Nrf2/HO-1 protein expression in the MTX-caused nephrotoxicity rat model. (**A**) Nrf2 and the corresponding β-actin bands of each group. (**B**) The diagram of the semi-quantified densitometric analysis of Nrf2 bands. (**C**) HO-1 bands and the corresponding β-actin bands of each group. (**D**) The diagram of the semi-quantified densitometric analysis. Data are displayed as means ± SEM. * and # mean a significant difference from the control group and MTX group, respectively, at *p* ≤ 0.05. ALO: Alogliptin, MTX: Methotrexate, Nrf2: Nuclear factor erythroid 2-related factor 2, HO-1: Heme oxygenase-1.

The protein expression of Nrf2 was significantly lower in rats challenged with MTX. However, pretreatment with ALO (20 mg/kg/day) remarkably upregulated Nrf2 expression (Figure 4A,B). Identically, heme oxygenase-1 (HO-1) expression in the control, ALO, MTX, and MTX + ALO groups elicited the same pattern. MTX significantly downregulated HO-1 relative to the control group, while ALO intervention significantly (*p* ˂ 0.05) increased the expression of the above-mentioned parameter (Figure 4C,D).

### 2.5. Effects of ALO on Renal Inflammatory Mediator (TNF-α) Protein Expression

**Figure 5:**
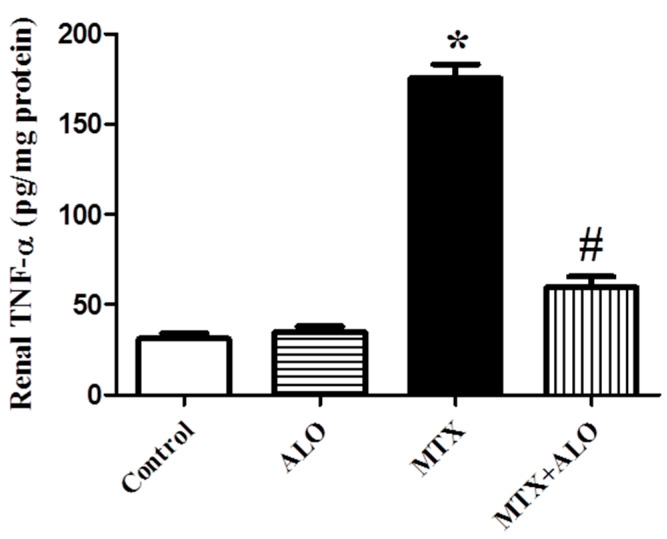
Effect of ALO (20 mg/kg) on renal TNF-α levels in the MTX-induced nephrotoxicity rat model. * and # mean a significant difference from the control group and MTX group, respectively, at *p* ≤ 0.05. ALO: Alogliptin, MTX: Methotrexate, TNF-α: Tumor necrosis factor-alpha.

There was a significant elevation in renal TNF-α, an inflammatory marker, level in rats following MTX intoxication. However, a significant decline in renal TNF-α was observed in the MTX + ALO group compared to untreated MTX rats, as presented in Figure 5.

### 2.6. Effect of ALO on the Renal C-Caspase-3 Protein Expression

**Figure 6:**
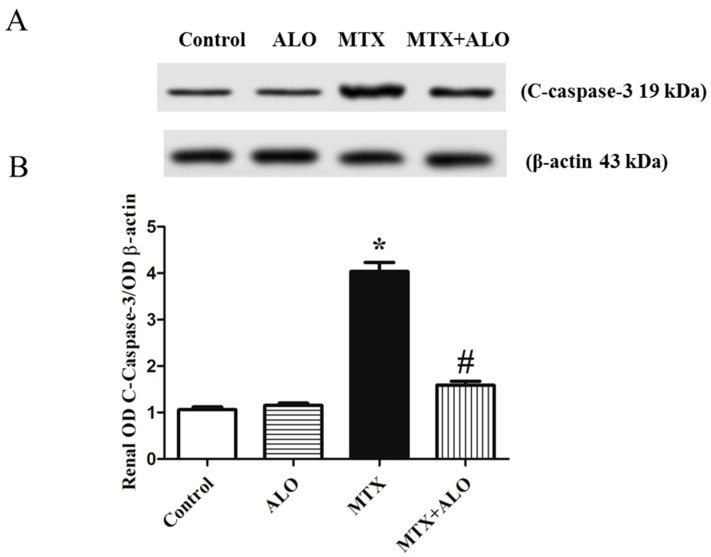
Representative Western blot illustrating the effect of ALO (20 mg/kg) on renal c-caspase-3 protein expression in the MTX-induced nephrotoxicity rat model. (**A**) C-caspase-3 and the corresponding β-actin bands of each group. (**B**) Representative semi-quantified densitometric analysis of c-caspase-3 bands. Data are displayed as means ± SEM. * and # mean a significant difference from the control group and MTX group, respectively, at *p* ≤ 0.05. ALO: Alogliptin, MTX: Methotrexate, C-caspase-3: Cleaved caspase-3.

Expression of cleaved caspase-3 (c-caspase-3), an apoptotic indicator, was investigated to explore the involvement of apoptosis in the pathogenesis of the MTX-produced renal dysfunction and to estimate the impact of abrogating apoptosis in defense against kidney damage. C-caspase-3 expression was significantly higher in the MTX-treated group than in the control rats. However, ALO produced a significant amelioration of the upregulated c-caspase-3 caused by MTX (Figure 6A,B).

## 3. Discussion

Although MTX is prevalently employed as an immunosuppressant and anticancer, hazards associated with its use, especially nephrotoxicity, remarkably restrict its clinical implementation. For the first time, the present study reported that the DPP-4 inhibitor, ALO, exerted potential protection against MTX-induced renal toxicity, confirmed by a marked attenuation in serum creatinine, urea, and renal KIM-1 mRNA expression alongside a notable enhancement in renal architecture. This therapeutic impact of ALO might be attributed to the restoration of oxidant-antioxidant imbalance, upregulated Nrf2/HO-1 pathway protein expression, and decline in both the inflammatory marker, TNF-α, and apoptotic one, c-caspase-3.

Here, animals intoxicated with MTX markedly showed greater levels of creatinine and urea, frequently measured renal physiological function indicators, besides elevated KIM-1 mRNA expression, referring to the negative impact of MTX on renal filtration and proximal tubules [39,40]. Several studies reported that the elevation in creatinine and urea levels reflected MTX-induced nephrotoxicity [41,42,43,44]. Furthermore, the increase in KIM-1, a tubular injury marker, was supported by Kutbi and Almalki [45], who reported the relation between elevated KIM-1 levels and the nephrotoxic impact of MTX on renal tubules. To validate these biochemical results, an examination of the renal structure was carried out. Notably, sections from the kidneys of the MTX group showed comparable histological injuries in the form of significant tubular degeneration and glomerular capillary dilatation. These outcomes are supported by earlier studies [46,47,48].

In contrast, ALO pretreatment restored the decline in renal performance and remarkably abrogated alteration in renal histology caused by MTX intoxication in harmony with previous publications that reported the potential of ALO to halt renal dysfunction [34,37,49]. Moreover, the ability of ALO to protect against cyclophosphamide-provoked tubular injury was reported [34]. According to these outcomes, molecular mechanisms underlying the renal protective impact of ALO in MTX settings were investigated.

Accumulating data pointed out that ROS plays a detrimental role in renal health impairment, causing damage to nephrons and altering the structures and functions of tubules and glomeruli [50]. Certainly, prior research has demonstrated that MTX acts as a ROS producer [51,52], depleting cellular levels of GSH and NADPH [53,54] and intensifying renal oxidative damage. Massive generation of ROS oxidizes the polyunsaturated phospholipids present in the cellular membrane, producing MDA, and exceeds the antioxidant tools such as GSH and CAT. GSH, an electrophile-neutralizing agent, scavenges toxic molecules such as ROS and reactive nitrogen species (RNS), involved in xenobiotic detoxification and redox signaling [55]. Hence, the impaired oxidant-antioxidant balance triggered by MTX was apparent in the present study by raised renal lipid peroxidation, MDA, and declined renal antioxidant defenses, indicated by decreased renal GSH levels and catalase activities consistent with prior reports [45,56].

As ROS accumulate, lipid peroxidation increases, which is an indicator of renal injury. Importantly, ALO administration hindered oxidative stress, as evidenced by a significant decrease in MDA levels that represents the lipid peroxidation degree alongside higher GSH concentrations and catalase capacities, proving the ability of ALO to scavenge radicals and protect against renal injury. These results confirm previously published studies that demonstrated the antioxidant property of DPP4 inhibitors and its relation to their defense mechanism in multiple experimental models of diabetic renal injuries [30,57], gentamicin [58], and antibody against the glomerular basement membrane [59].

Worthily, MTX-triggered disturbance in renal oxidative status could be explained by modulation of Nrf2 and HO-1, a downstream target of Nrf2 [60,61]. Nrf2, a leading transcription factor present in many organs, including the kidney, controls the expression of proteins related to the defense against oxidation and inflammation [62,63,64,65]. Also, a cascade of heme breakdown initiated by HO-1 produces chemicals that provide protection against oxidative hazards in various illnesses, including renal ailments [66]. Based on prior studies that highlighted the possible protective impact of the Nrf2/HO-1 pathway against the onset and progression of MTX-induced toxicity [47,67], the expressions of Nrf2 and HO-1 were assessed using Western blotting in all groups. Renal tissues challenged with MTX showed a significant suppression in this protective pathway, consistent with the results of MDA, catalase, and GSH. However, ALO preconditioning resulted in a significant increase in expression of cytoprotective Nrf2 and HO-1, protecting kidney cells from oxidative damage caused by MTX, supporting the previous publications highlighting the DPP4 inhibitor-enhanced Nrf2/HO-1 pathway and its positive impact against organ damage [37,68,69]. Therefore, the antioxidant influence of ALO in MTX renal dysfunction could be explained by the enhancement of the endogenous Nrf2/HO-1 signaling pathway.

It is worth mentioning that MTX-induced inflammation is mainly triggered by oxidative stress, which eventually activates nuclear factor-kappa B (NF-κB), a key factor of redox response [70]. In turn, activation of NF-κB leads to transcription of inflammatory markers such as TNF-α, which worsens inflammatory responses via encouraging immune cell infiltration and eventual cell death [5,70]. Hereby, the boost in renal inflammatory marker, TNF-α, confirmed the contribution of inflammatory consequences in MTX-triggered renal damage as discussed previously [10,71]. Of particular importance, treatment with ALO mitigated the increase in TNF-α level resulting from MTX intoxication, referring to the anti-inflammatory potential of ALO, which partially justifies its renoprotective influence. Several studies shed light on the anti-inflammatory activity of DPP4 inhibitors and their implication in defense against organ destruction [68,72,73].

There is a growing attention to the possible role of Nrf2 activation in ameliorating inflammation, triggering anti-inflammatory signaling, and attenuating pro-inflammatory gene expression [10,74]. Nrf2 binds the promoter regions of the genes encoding inflammatory cytokines and suppresses RNA polymerase II attachment to DNA, leading to the abrogation of their transcription [75]. Of interest, Nrf2 deficiency elevates NF-κB expression, a key regulator of inflammatory reactions, leading to increased generation of inflammatory factors such as TNF-α. Meanwhile, lipopolysaccharide-induced transcription of pro-inflammatory cytokines was prevented by Nrf2 activation [76]. Here, the anti-inflammatory effect of ALO was proven by a remarkable decline in renal TNF-α level, which might be due to the augmentation of antioxidant defense machinery and Nrf2/HO-1 signaling. Botros, Matouk [37] also discussed the interplay between Nrf2 and inflammatory markers, NF-κB and TNF-α, and their contribution to renal protection caused by ALO.

Furthermore, excessive production of ROS and pro-inflammatory cytokines often drives mitochondrial apoptotic pathways [54]. Therefore, the renal expression of c-caspase-3 was investigated in the four groups to examine the role of apoptosis in MTX-induced renal destruction and the possible antiapoptotic role of ALO. A significant increase in c-caspase-3 expression has been observed in MTX-intoxicated rats in harmony with perturbed renal structure manifested as necrosis of the epithelial lining. Earlier studies showed MTX causes apoptosis in several organs, including renal tissues [77,78], hepatic tissues [79,80], and heart tissues [81,82]. It has been demonstrated that MTX-induced renal cell death is mediated via autophagy-related proteins and apoptosis. Notably, when ALO was given as a pretreatment, apoptosis was attenuated, evidenced by a decline in caspase-3 expression. Consistent with earlier studies, DPP4 inhibitors have been found to exert a protective effect through antiapoptotic signaling pathways in various models of organ toxicity, such as diabetic renal complications [30], cyclophosphamide-induced either hepatotoxicity [83], nephrotoxicity [34], or lung [84], and doxorubicin-induced testicular toxicity [72] or nephrotoxicity [37]. Hence, the antiapoptotic effects of ALO may partially mediate its protection against MTX-induced nephrotoxicity.

Overall, we examined, for the first time, the nephroprotective effect of ALO in an experimental MTX model of nephrotoxicity. The attenuation of oxidative stress, inflammation, and apoptosis by ALO contributed to its nephroprotective effects. Renoprotective effects of ALO in our model cannot be attributed to changes in blood glucose level as all values were within or close to the reported physiological ranges and differences were not statistically significant [85]. Together, these results suggest that ALO is a promising therapy that limits kidney injury in patients receiving MTX.

However, assessment of DPP-4 level and its relation to MTX-induced nephrotoxicity is a limitation of the present study that should be the focus of our future research. Further clinical studies should be considered before clinical application to examine whether experimental findings could be translated to human subjects and to explore the safety and optimal dosing regimen of this drug. Long-term investigations should also be performed to assess the effects of extended alogliptin exposure on renal function and the sustainability of its nephroprotection.

## 4. Materials and Methods

### 4.1. Drugs and Chemicals

Methotrexate and ALO were obtained from Hauptpharma GmbH, Wolf-Ratshausen, Germany, and Hikma Pharmaceuticals, London, UK, respectively. Colorimetric diagnostic kits for urea, creatinine, and MDA were bought from the Biodiagnostic Company, Cairo, Egypt. For Western blotting, primary antibodies against the investigated proteins Nrf2 and HO-1 were purchased from Santa Cruz Biotechnology, Dallas, TX, USA, with catalog numbers sc-365949 and sc-136960. Meanwhile, c-caspase-3 antibody was bought from Cell Signaling Technology, Danvers, MA, USA, with Catalog No. 9661. The Rat TNF-α ELISA kit from BioLegend’s ELISA MAX™ Deluxe, San Diego, CA, USA, with Catalog No. 438204, was used.

### 4.2. Experimental Design

Forty adult *Wistar* male rats with weights ranging from 180 to 200 g were used. They were purchased from the faculty of Pharmacy at Nahda University in Beni Suef, Egypt. The rats were allocated in a controlled environment with a humidity level of 50 ± 10% and a temperature of 21 ± 2 °C. The rats were also subjected to a 12 h cycle of light and dark and provided with unlimited ordinary commercial food and water to guarantee that they were properly nourished. Following two weeks of adaptation, the animals were randomly distributed into four groups with an independent investigator. Each group consists of ten rats treated as follows:

1. The control group was given the vehicle of ALO, 0.5% carboxymethyl cellulose (CMC), orally for ten consecutive days and a single dose of saline via intraperitoneal route on the seventh day.
2. The ALO group was orally administered ALO at a dosage of 20 mg/kg/day for ten days while being intraperitoneally injected with saline on the 7th day.
3. The MTX group received 0.5% CMC orally for ten days and was exposed to a single intraperitoneal injection of MTX at a dosage of 20 mg/kg on day 7.
4. The MTX + ALO group was given ALO orally at a dose of 20 mg/kg/day for ten days while receiving an intraperitoneal injection of MTX (20 mg/kg) on day 7 of the experimentation period.

The dose of MTX was selected to be adequate to elicit renal impairment [46,86]. Meanwhile, the dose of 20 mg/kg/day for ALO was selected based on our preliminary studies and earlier pharmacological studies reporting its pronounced protective potential in different rodent models of organ injury such as cyclophosphamide-induced renal [34], hepatic [83] and pulmonary [84] impairment and doxorubicin-induced nephrotoxicity [37].

### 4.3. Tissue and Blood Collection

On the eleventh day, total rat body weights were recorded, and blood specimens were collected from the rats’ tails to measure the level of random blood glucose using an on-call plus glucometer.

Afterward, animals were fully anesthetized by pentobarbital sodium (50 mg/kg, I.P.). Then, the left ventricle was punctured to withdraw blood, followed by immediate decapitation [87] to minimize distress. The collected blood was exposed to centrifugation for a duration of 15 min at a speed of 5000 rpm to separate clear, fresh serum in order to measure renal performance indices.

For sample tissue collection, the rats underwent a surgical abdominal incision. Both kidneys were promptly removed and weighed to calculate the kidney index by dividing both kidneys’ weight by the corresponding body weight and then multiplying by 100 [88]. The right kidney was homogenized in ice-cold phosphate buffer. The resultant homogenate was centrifuged at 3000 rpm for a third of an hour. Careful dividing of the supernatant into aliquots was carried out to hinder repeated thawing and re-freezing. They were preserved at a temperature of −80 °C. The left kidneys were immersed in buffered formalin solution (10%) for later histological analysis.

### 4.4. Determination of Kidney Function Biomarkers

For the detection of renal function, creatinine and urea levels in freshly isolated serum were quantified according to methods outlined by Schirmeister [89] and Fawcett and Scott [90], respectively.

### 4.5. Assessment of KIM-1 mRNA Expression by Quantitative Real-Time (RT)-PCR

The SV Total RNA Isolation method was conducted to extract total RNA from the homogenized renal tissue in accordance with the recommendations of the manufacturer (Thermo Scientific, Waltham, MA, USA). For further purification and elimination of any remaining genomic DNA, the extracted RNA was subjected to RNase-free DNase. Extracted RNA yield [1 μg] was converted into a single cDNA strand with the aid of a cDNA reverse transcription kit (Thermo Fisher Scientific, USA). A digitally programmed Primer Express 2.0 (Applied Biosystems, Foster City, CA, USA) aided in the design of KIM-1- and GAPDH-specific primers. The sequences of primers were found in Table 3.

### 4.6. Histopathological Examination of Renal Tissues

The formalin-fixed renal tissues were cut, neatened, dehydrated in alcohol, and washed in xylene. Then, they were subjected to embedding in paraffin and trimmed into slices of 4–6 μm thick. Hematoxylin and eosin (H&E)-stained renal specimen [92]. The usual histopathological investigation was performed with the aid of a light digital microscope (Olympus XC30, Tokyo, Japan).

Changes in the kidney morphological structure were graded using the scoring system of Endothelial, Glomerular, Tubular, and Interstitial (EGTI) [93].

### 4.7. Determination of Renal Oxidative Stress Parameters

MDA, an indicator of lipid peroxidation, was determined in the unit of nmol/mg tissue protein by employing a specialized method described by Buege and Aust [94]. Furthermore, GSH content and catalase activity, representing endogenous antioxidant defense, in renal homogenates were measured dependent on the procedures reported by Ellman [95] and Greenwald [96] and expressed as nmol/mg tissue protein and U/mg tissue protein, respectively.

### 4.8. Nrf2, HO-1, and C-Caspase-3 Assessment Using Western Blot Analysis

Renal protein expressions involved in the Nrf2/HO-1 pathway and c-caspase-3 of all studied groups were investigated using Western blotting techniques. The protein extraction kit was used to extract total protein from the homogenized frozen renal tissue samples (Bio Basic Inc., Markham, ON, Canada). With the aid of the Bradford Protein Assay Kit from Bio Basic Inc., the protein concentration was assessed in each specimen. Depending on the molecular weight of proteins, they were separated by loading equal protein amounts (20 µg) on sodium dodecyl sulfate-polyacrylamide gel electrophoresis (SDS-PAGE). Then, they were electro-transferred to polyvinyl difluoride (PVDF) membranes. Incubation in TBST buffer containing 3% BSA at room temperature for 1 h was performed to mitigate nonspecific binding of the membrane. Then, incubation of membranes with primary antibodies specific to the investigated target proteins, Nrf2 (dilution 1:1000), HO-1 (dilution 1:1000), and c-caspase-3 (dilution 1:1000) was performed at 4 °C overnight.

After multiple washings of the membranes using TBST, the membranes were immersed in a secondary antibody conjugated to horseradish peroxidase (HRP) at room temperature for an hour. The Clarity TM Western ECL substrate, a chemiluminescent substrate (Bio-Rad with Catalog No. 170-5060), was employed to visualize the bands. The produced bands were captured using a CCD camera-based imager. Then, the band densities of proteins of interest were quantified and analyzed by ChemiDoc MP imaging system (version 3) (Bio-Rad Inc., Hercules, CA, USA) against those of their corresponding β-actin.

### 4.9. Determination of Renal TNF-α Protein Expression

The levels of TNF-α were evaluated based on the sandwich ELISA principle employing the instructions of the kit manufacturer.

### 4.10. Statistical Analysis

The findings were expressed as mean ± standard error of the mean (SEM) and statistically checked by one-way analysis of variance (ANOVA) test. Tukey–Kramer post hoc test was performed to compare different groups. In the case of a probability (P) value less than 0.05, the results are considered statistically significant. Graph Pad Prism^®^ software (Version 5.0, GraphPad Software, San Diego, CA, USA) was utilized to undergo these statistical tests.

## 5. Conclusions

Regarding this study results, it is proposed that ALO may provide preservation against MTX-induced kidney damage. This renoprotective influence elicited by ALO can be attributable to launching the Nrf2/HO-1 signaling pathways. In addition, the mitigation of inflammation by suppressing TNF-α and apoptosis by declining c-caspase-3 may also be involved. These findings draw attention to the need for more extensive clinical research to determine whether ALO may shield cancer patients receiving MTX therapy from renal damage.

## Figures and Tables

**Table 1:** Effect of ALO (20 mg/kg) on glycemic level, renal index, serum creatinine, and urea concentration in MTX-induced renal damage.

Groups	Random Blood Glucose Level (mg/dL)	Kidney Index	Serum Creatinine Level (mg/dL)	Serum Urea Level (mg/dL)
Control	120.50 ± 7.18	0.51 ± 0.01	0.86 ± 0.02	29.69 ± 1.50
ALO	129.30 ± 7.60	0.53 ± 0.01	0.89 ± 0.04	31.25 ± 1.55
MTX	150.20 ± 9.15	0.64 ± 0.03 *	4.00 ± 0.18 *	62.33 ± 1.97 *
MTX + ALO	138.20 ± 8.61	0.52 ± 0.02 #	0.82 ± 0.06 #	46.56 ± 3.04 #

Data are displayed as means ± SEM (*n* = 6). * and # mean a significant difference from the control and MTX group, respectively, at *p* ≤ 0.05. ALO: Alogliptin, MTX: Methotrexate.

**Table 2:** The Endothelial, Glomerular, Tubular, and Interstitial (EGTI) histology scoring system for kidney lesions.

	Lesion	Tubular	Glomerular	Tubulo/Interstitial
Groups				
Control		0	0	0
ALO		0	0	0
MTX		3	3	3
MTX + ALO		1	1	0

A score level of (0) means no damage, a score level of (1) refers to the presence of histopathological abnormalities in less than 25% of the inspected tissue, and a score level of (3) means the appearance of morphological alterations in up to 60% of the examined field. ALO: Alogliptin, MTX: Methotrexate.

**Table 3:** Sequences of the primers.

	Forwarded	Reverse	
KIM-1	5′-TTCAGATGTGCTGCTGCTGT-3′	5′-AAGGGAAAGGCTGGCAAGTC-3′	NM_001003336.2
GAPDH	5′-CATCACTGCCACCCAAAGACTG-3′	5′-TGCCAGTGAGCTTCCCGTTCAG-3	NM_008084

Amplification and detection were performed by the 7900 HT Real Time PCR System (Applied Biosystems) and SYBR Green emission (SYBR Green Master Mix, Applied Biosystems). The relative expression of the target gene depending on the mean critical threshold (CT) of GAPDH, the housekeeping gene was analyzed and presented as 2^−ΔΔCT^ [91].

## Data Availability

All data generated or analyzed during this study are included in this published article.

## Abbreviations

The following abbreviations are used in this manuscript:

MTX	Methotrexate
ALO	Alogliptin
Nrf2	Nuclear factor erythroid 2-related factor 2
HO-1	Heme oxygenase-1
AKI	Acute kidney injury
ROS	Reactive oxygen species
GLP-1	Glucagon-like peptide 1
EGTI	Endothelial, Glomerular, Tubular, and Interstitial
RNS	Reactive nitrogen species
NF-κB	Nuclear factor-kappa B
Cr	Creatinine
MDA	Malondialdehyde
CMC	Carboxymethyl cellulose
GSH	Reduced glutathione
H&E	Hematoxylin and eosin
